# Dissemination of *Chlamydia* from the reproductive tract to the gastro-intestinal tract occurs in stages and relies on *Chlamydia* transport by host cells

**DOI:** 10.1371/journal.ppat.1008207

**Published:** 2019-12-02

**Authors:** Savannah E. Howe, Nita Shillova, Vjollca Konjufca

**Affiliations:** School of Biological Sciences, Microbiology Program, Southern Illinois University, Carbondale, Illinois, United States of America; Duke University School of Medicine, UNITED STATES

## Abstract

*Chlamydia trachomatis* is a Gram-negative bacterial pathogen and a major cause of sexually transmitted disease and preventable blindness. In women, infections with *C*. *trachomatis* may lead to pelvic inflammatory disease (PID), ectopic pregnancy, chronic pelvic pain, and infertility. In addition to infecting the female reproductive tract (FRT), *Chlamydia* spp. are routinely found in the gastro-intestinal (GI) tract of animals and humans and can be a reservoir for reinfection of the FRT. Whether *Chlamydia* disseminates from the FRT to the GI tract via internal routes remains unknown. Using mouse-specific *C*. *muridarum* as a model pathogen we show that *Chlamydia* disseminates from the FRT to the GI tract in a stepwise manner, by first infecting the FRT-draining iliac lymph nodes (ILNs), then the spleen, then the GI tract. Tissue CD11c^+^ DCs mediate the first step: FRT to ILN *Chlamydia* transport, which relies on CCR7:CCL21/CCL19 signaling. The second step, *Chlamydia* transport from ILN to the spleen, also relies on cell transport. However, this step is dependent on cell migration mediated by sphingosine 1-phosphate (S1P) signaling. Finally, spleen to GI tract *Chlamydia* spread is the third critical step, and is significantly hindered in splenectomized mice. Inhibition of *Chlamydia* dissemination significantly reduces or precludes the induction of *Chlamydia*-specific serum IgG antibodies, presence of which is correlated with FRT pathology in women. This study reveals important insights in context of *Chlamydia* spp. pathogenesis and will inform the development of therapeutic targets and vaccines to combat this pathogen.

## Author summary

*Chlamydia trachomatis* is a bacterial pathogen and a major cause of sexually transmitted disease and preventable blindness worldwide. In women, *C*. *trachomatis* may cause PID, ectopic pregnancy, chronic pelvic pain, and infertility. *Chlamydia* spp. are routinely found in the gastro-intestinal (GI) tract of humans and animals. However, whether and how *Chlamydia* spreads internally to the GI tract following the female reproductive tract (FRT) infection remains unknown. Using a mouse model of infection here we show that *Chlamydia* spreads to the GI tract in a stepwise manner, by first infecting the FRT-draining iliac lymph nodes (ILNs), then the spleen and the GI tract. Tissue DCs mediate the first step: FRT to ILN *Chlamydia* spread, which relies on CCR7:CCL21/CCL19 signaling. The second step, ILN to spleen spread, also relies on cell migration, and is dependent on sphingosine 1-phosphate (S1P) signaling. Finally, spleen to GI tract *Chlamydia* spread is the third critical step and is significantly hindered in splenectomized mice. Our study reveals important insight in context of *Chlamydia* pathogenesis. In addition, this work will inform the identification of therapeutic targets and development of vaccines against this pathogen.

## Introduction

Sexually transmitted infections (STIs) remain a major health challenge worldwide. Recently WHO estimated that about 1 million new STIs are acquired daily. In 2016, about 376 million new STIs were reported world-wide, of which 127 million were caused by *Chlamydia* [1]. In the US, *Chlamydia* spp. continue to be the leading cause of STIs, representing 1.7 million cases of approximately 2.3 million STIs reported in 2017 [2]. The highest rates of new chlamydial infections occur in young adults, especially young women of reproductive age. In about 70–80% of cases, chlamydial infections in women are asymptomatic and as such may go untreated. About 15% of untreated chlamydial infections progress to PID [3–5]. If left untreated, PID may result in infertility, ectopic pregnancy, and chronic pelvic pain [6]. Pregnant women infected with *Chlamydia* can pass the infection to their infants, potentially resulting in neonatal ophthalmia and pneumonia. Infection with *Chlamydia* also increases the risk for HIV transmission and HPV-associated cervical cancer [7, 8]. With specific regard to *Chlamydia*, to date no effective vaccines have been licensed for use in humans. *Chlamydia* spp. are obligatory intracellular bacterial pathogens that undergo a biphasic developmental cycle, thus they exist in two forms: the infectious elementary bodies (EBs) and the non-infectious reticulate bodies (RBs) [9]. Host infection is initiated when EBs first infect epithelial cells that line mucosal surfaces (e.g. FRT). Within epithelial cells, EBs enclose themselves within an endocytic vesicle termed inclusion, where EBs transform into RBs. Within an inclusion, RBs divide by binary fission and then transform back into the infectious EB form. EBs are released following cell lysis or inclusion extrusion, and then go on to infect other neighboring cells. *C*. *muridarum* is a mouse-specific pathogen and does not infect humans, although it shares near genomic synteny with human-specific *C*. *trachomatis* [10]. However, *C*. *muridarum* has been widely used as a model to study both pathogenesis of and immunity to human-specific *C*. *trachomatis*. This is because intravaginal infections of mice with *C*. *muridarum* mimic *C*. *trachomatis* pathology in humans, which may result in hydrosalpinx and infertility [11–14]. Human-specific *C*. *trachomatis* strains have been used for per-vaginal (PV) infections of mice. However, such infections do not recapitulate the pathology observed in humans as they cause a mild, short-lived FRT infection and no post-infection upper FRT pathology [15]. Human specific chlamydial strains produce upper FRT pathologies in mice only when animals are inoculated with large doses of *Chlamydia* directly in the uterus or ovaries [16], which does not represent physiological conditions of human infection.

In addition to infecting the FRT, in both humans and animals, *Chlamydia* spp. infect and persist in the GI tract without causing inflammation or pathology. GI tract *Chlamydia* are more resistant to azithromycin treatment and represent a potential reservoir for recurring FRT infections [17–19]. Treatment failure rates for rectal *Chlamydia* in men and women range between 6% and 23% [20, 21]. In humans, *C*. *trachomatis* infection of the GI tract may occur via oral or anal intercourse however, it remains unknown whether *C*. *trachomatis* disseminates from the FRT to the GI tract internally and independent of sexual behavior. In mice, following FRT infection *C*. *muridarum* reaches the GI tract via an internal, non-oral, non-rectal route [22]. Detection of *C*. *muridarum* genomes in the FRT, GI tract, liver, spleen, heart, and lung 4 weeks after infection, indicates a systemic spread of the pathogen [22]. Moreover, when administered intravenously (IV) *C*. *muridarum* infects the GI tract [23] and its genome copies are detected systemically for up to 14 d after IV infection, suggesting that *C*. *muridarum* survives in the circulation. However, live *Chlamydia* was not detected systemically after day 7 of infection, which coincided with the peak of GI tract live *Chlamydia* loads, leading authors to suggest a temporal correlation between blood-borne and GI tract *Chlamydia* [23].

Considering that *Chlamydia* spp. are obligate intracellular pathogens we hypothesized that following FRT infection *Chlamydia* spp. rely on cell transport in order to reach the GI tract. Here we confirm our hypothesis and show that inhibition of cell migration abrogates *Chlamydia* spread from the FRT to the GI tract. In our experimental model in which coprophagy and grooming are precluded, we show that *Chlamydia* infection of the spleen is a critical step for *Chlamydia* dissemination to the GI tract. Finally, we show that carriage of *Chlamydia* by CD11c^+^ DCs from the FRT to the draining ILNs represents the first step in *Chlamydia* systemic dissemination. This work will improve our understanding of the mechanisms that govern FRT to ILN cell migration and antigen transport. Further elucidating the mechanisms of and the importance of *Chlamydia* transport and systemic dissemination in long-term FRT pathology will be critical for the development of vaccination and therapeutic approaches.

## Results

### Dissemination of *Chlamydia* from the FRT to the GI tract occurs in stages

**Fig 1:**
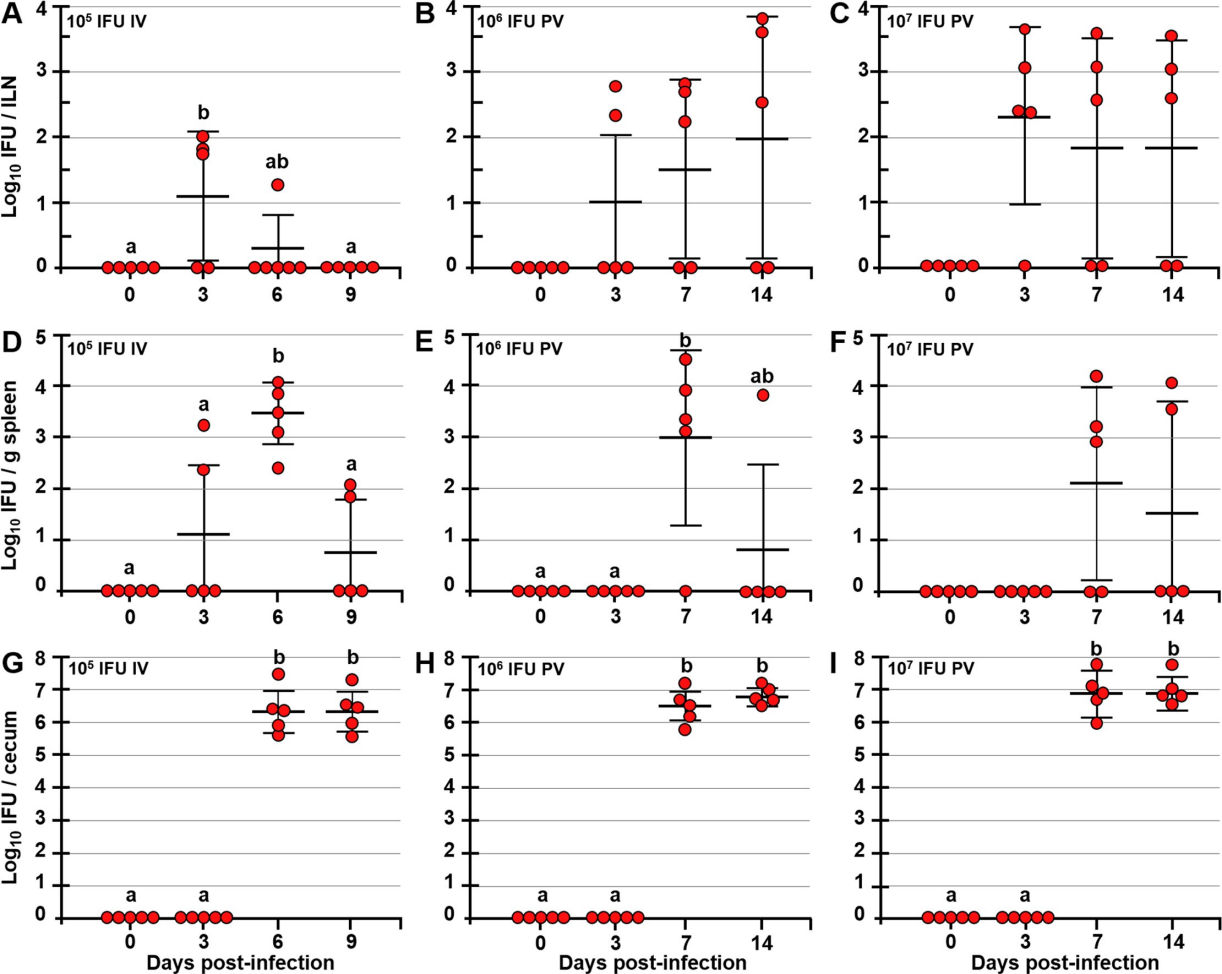
Following IV and PV infection *Chlamydia* reaches the ILNs, spleen and the GI tract of C57BL/6 mice. *Chlamydia* titers in ILNs (A-C), spleens (D-F) and ceca (G-I) of mice at 0, 3, 6, 9 or 0, 3, 7, 14 days post IV or PV infections, respectively. *Chlamydia* titers are expressed as log_10_ number of IFU per ILNs, per g of spleen, or per cecum. Data are expressed as the mean ± SD. Group means were separated using Tukey’s multiple comparison test and declared significantly different at p<0.05 (n = 5 mice per time point). Group means that do not share superscript are significantly different (p<0.05).

In female mice, PV infection with *C*. *muridarum* causes tubal fibrosis and hydrosalpinx pathology similar to long-term disease sequelae caused by *C*. *trachomatis* in humans. For these reasons, the mouse PV model of infection is extensively used. In addition, in both humans and mice *C*. *trachomatis* and *C*. *muridarum* respectively, infect the GI tract without causing pathology. Whether and how *Chlamydia* spp. may spread internally remains unknown. However, others have suggested that in mice *Chlamydia* spreads from the FRT to the GI tract via blood circulation [23]. Considering that *C*. *muridarum* (like *C*. *trachomatis*) is an intracellular pathogen, we hypothesized that its dissemination from the FRT to the GI tract depends on cell transport. To begin addressing this hypothesis, C57BL/6 mice were PV infected with 10^3^, 10^4^, 10^5^, 10^6^ or 10^7^ inclusion forming units (IFU) of *C*. *muridarum*. At 0, 3, 7 and 14 days post-infection (dpi), ILNs, spleen, liver, and cecal scrapings were collected for determination of *Chlamydia* titers. In a set of studies mice were infected IV with 10^5^ IFU of *C*. *muridarum* in order to investigate the dynamics of systemic *Chlamydia* clearance. To preclude GI tract infection via grooming or coprophagy, PV-infected mice were singly housed on wire-mesh bottom cages and fitted with lightweight neck collars. In IV-infected mice, live *Chlamydia* was detected in ILNs by 3 dpi and was cleared by 9 dpi (Fig 1A). As expected, IV administered *Chlamydia* was captured in the spleen, reached peak titer by 6 dpi and was mostly cleared by 9 dpi (Fig 1D). In contrast, PV-infected mice had high *Chlamydia* ILN titers even at 14 dpi, possibly indicating a delayed clearance due to a continuous *Chlamydia* supply to the ILN from the infected FRT (Fig 1B and 1C). In PV-infected mice, *Chlamydia* was detected in the spleen by 7 dpi regardless of the dose of infection (10^3^−10^7^ IFU), (Fig 1E and 1F and S1B Fig). However, it is important to note that mice PV infected with 10^3^, 10^4^ or 10^5^ IFU of *Chlamydia* exhibited lower ILN, spleen, and cecal titers compared to mice infected with 10^6^ or 10^7^ IFU of *Chlamydia* (S1A–S1C Fig). In addition, ceca of 2/5, 2/5 and 1/5 mice infected with 10^3^, 10^4^, or 10^5^, respectively, were negative for *Chlamydia* at 7 dpi (S1C Fig). This finding indicates that low dose may delay, but not preclude *Chlamydia* systemic spread. By 14 dpi, most animals cleared *Chlamydia* from the spleen and *Chlamydia* clearance was slower in mice infected with higher doses (Fig 1E and 1F). Finally, in all infected mice (IV or PV) *Chlamydia* colonized the ceca by 7 dpi, and at high PV doses (10^6^ and 10^7^ IFU) 100% of mice had cecal *Chlamydia* titers at 7 dpi, which were not significantly different from cecal titers observed at 14 dpi (Fig 1G–1I). To ensure that animals were infected with the intended dose of *Chlamydia*, vaginal swabs were collected and titered (S1D Fig). In addition, all mice were infected from one common *Chlamydia* stock that was prepared and titered in advance. Unlike some other bacterial pathogens (e.g. *Salmonella* spp.), *Chlamydia* does not infect the liver, as only 1/80 PV-infected mice had detectable *Chlamydia* titers in the liver, regardless of the dose of PV infection (S1E Fig). Similarly, no *Chlamydia* was found in gallbladder/bile, plasma, or blood cell fractions tested at different times after PV infection (S1E Fig). *Chlamydia* tissue titers shown here are from the first passage. Although all samples were passaged 6 times, all negative samples remained negative even after 6 passages. These data indicate that *Chlamydia* disseminates systemically in a stepwise manner, by first infecting the FRT-draining ILNs (by 3 dpi), then the spleen, and the GI tract (by 7 dpi).

### CCR7-mediated cell migration is important, but not essential for *Chlamydia* dissemination to the ILN, spleen, and the GI tract

**Fig 2:**
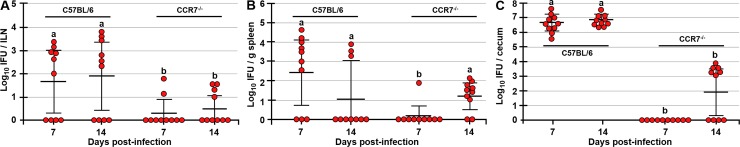
CCR7 signaling is important but not essential for *Chlamydia* dissemination to the ILNs, spleen, and the GI tract. *Chlamydia* titers in ILNs (A), spleen (B) and ceca (C) of C57BL/6 (control) and CCR7^-/-^ mice at 7 and 14 dpi PV with 10^6^ IFU of *Chlamydia*. *Chlamydia* titers are expressed as log_10_ number of IFU per ILNs, per g of spleen, or per cecum. Data are representative of two experiments and are expressed as the mean ± SD. Group means were separated using Student’s t-test (n = 10 mice per time point). Group means that do not share a superscript are significantly different from each other (p<0.05).

Following an infection, tissue DCs capture the antigen, carry it to the lymph nodes and present it to T cells for induction of adaptive immune responses. Upon pathogen encounter tissue DC (e.g. skin DC) mature, upregulate the expression of CD80/CD86 and the chemokine receptor CCR7 [24, 25]. The interaction of CCR7 with CCL21/CCL19 ligands mediates DC migration to local draining lymph nodes, where DCs present captured antigens to T cells, thus activating adaptive immunity [24–27]. CCL21 and CCL19 are expressed in both lymphatic endothelial cells and lymph nodes [28], therefore the CCR7:CCL21/CCL19 axis is essential for intravasation of tissue DCs into lymphatics and for DC migration to local draining lymph nodes. To investigate the role of CCR7-mediated cell migration in *Chlamydia* dissemination, CCR7^-/-^ and C57BL/6 mice (controls) were PV infected with 1x10^6^ IFU of *Chlamydia*. We chose to use a higher dose, since this dose allows for 100% infection of the GI tract by 7 dpi. We reasoned that if *Chlamydia* dissemination is inhibited at a high dose of PV infection, the inhibition would be more drastic for lower infectious doses. We found that systemic dissemination of *Chlamydia* was significantly inhibited in CCR7^-/-^ mice, resulting in reduced titers in ILN (p<0.01), spleen (p<0.01), and ceca (p<0.01) at 7 dpi compared to C57BL/6 controls (Fig 2). At 14 dpi ILN and cecal *Chlamydia* titers in CCR7^-/-^ mice were significantly lower compared to 14 dpi controls (p<0.02 and p<0.001, respectively). However, there were no differences in spleen titers between CCR7^-/-^ and C57BL/6 mice at 14 dpi (p<0.5) (Fig 2B). Overall this data shows that CCR7:CCL19/CCL21 signaling axis is important for *Chlamydia* dissemination, and that the delayed *Chlamydia* spread to the ILNs is likely due to impaired CCR7-mediated cell migration. In addition, the delayed infection of the ILN, spleen, and the GI tract in CCR7^-/-^ mice indicates that in the absence of CCR7, signaling via other receptor/chemokine pairs such as CCR8:CCL1/CCL8, CXCR4/CXCL12, CX3CR1/CX3CL1 and/or S1PR: S1P might direct cell migration and thus allow for *Chlamydia* systemic dissemination, albeit less efficiently.

### Inhibition of S1P-mediated cell migration by FTY720 inhibits *Chlamydia* transport to the spleen and the GI tract, but not to the FRT-draining ILNs

**Fig 3:**
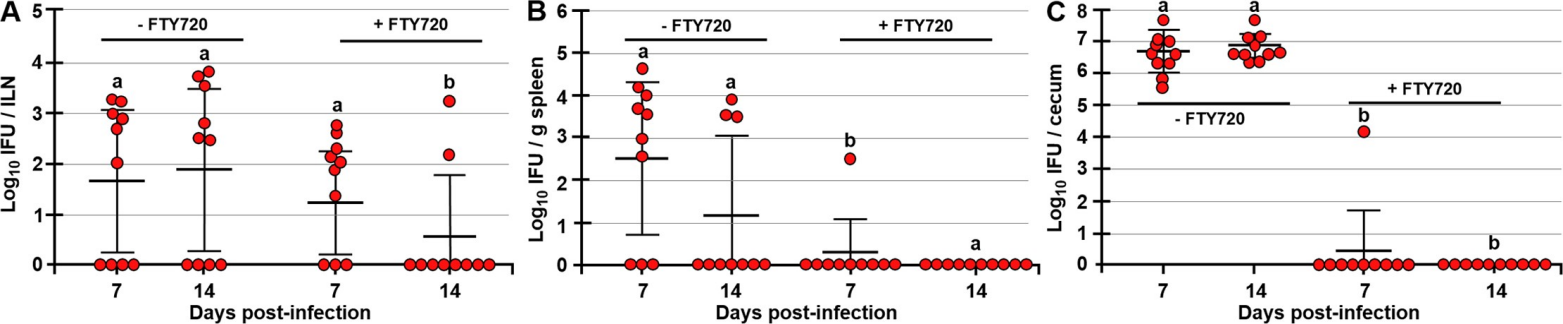
Inhibition of S1P signaling by FTY720 abrogates cell egress from the ILNs and *Chlamydia* dissemination to the spleen and the GI tract. *Chlamydia* titers in ILNs (A), spleen (B), and ceca (C) of C57BL/6 (control) and FTY720-treated mice at 7 and 14 dpi PV with 10^6^ IFU of *Chlamydia*. *Chlamydia* titers are expressed as log_10_ number of IFU per ILNs, per g of spleen, or per cecum. Data are representative of two experiments and are expressed as the mean ± SD. Group means were separated using Student’s t-test (n = 10 mice per time point). Group means that do not share a superscript are significantly different from each other (p<0.05).

In addition to CCR7, mature DC upregulate the expression of S1P receptors (S1PR) which allows them to migrate by sensing higher S1P concentrations in lymph and blood [29]. In vivo, S1P-mediated cell migration can be inhibited by FTY720, an S1P analog [30]. We reasoned that if S1P mediates the FRT to ILN and ILN to spleen cell migration, *Chlamydia* would not disseminate systemically. To test this hypothesis, FTY720 was provided to mice via drinking water for the duration of the studies, starting at 3 days prior to PV infection with 10^6^ IFU of *Chlamydia*. Interestingly, S1P signaling does not appear to be essential for FRT-ILN cell migration as *Chlamydia* ILN titers of FTY720-treated mice were not significantly different from controls at 7 dpi (p<0.2) (Fig 3A). However, S1P signaling is essential for cell egress from the ILNs and thus for *Chlamydia* transport to the spleen and the GI tract (Fig 3B and 3C). Compared to controls, FTY720-treated mice had significantly reduced *Chlamydia* loads in the spleen (p<0.002 and p<0.06) and ceca (p<0.001 and p<0.001) at 7 and 14 dpi, respectively (Fig 3B and 3C).

### Infection of the spleen is important for *Chlamydia* dissemination to the GI tract

**Fig 4:**
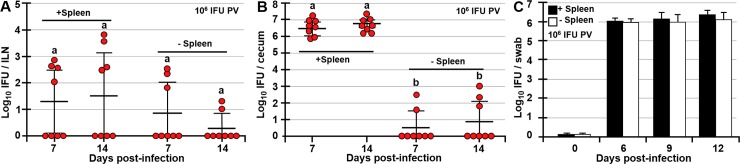
Infection of the spleen by *Chlamydia* is a key step for its dissemination to the GI tract. *Chlamydia* titers in ILNs (A) and ceca (B) of C57BL/6 (control) and splenectomized mice at 7 and 14 dpi PV with 10^6^ IFU of *Chlamydia*. (C) *Chlamydia* titers in vaginal swabs of C57BL/6 (control) and splenectomized mice at 0, 6, 9 and 12 dpi PV with 10^6^ IFU of *Chlamydia*. *Chlamydia* titers are expressed as log_10_ number of IFU per ILNs, per cecum, or per vaginal swab. Data are representative of two experiments and are expressed as the mean ± SD. Group means were separated using Student’s t-test (n = 8 mice per time point). Group means that do not share a superscript are significantly different from each other (p<0.05).

The results of CCR7^-/-^ and FTY720-treated mice show that CCR7 signaling is more important for the FRT-ILN cell migration and thus the first step of *Chlamydia* transport, while S1P signaling is indispensable for cell egress from ILNs and therefore *Chlamydia* transport from the ILN to the spleen and the GI tract. We then hypothesized that infection of the spleen is the second important step for *Chlamydia* dissemination to the GI tract. To test this hypothesis splenectomized and control mice were PV infected with 10^6^ IFU of *Chlamydia*. We found that removal of the spleen significantly diminished GI tract infection by *Chlamydia* at 7 and 14 dpi (Fig 4B). Although ceca of 5/16 splenectomized mice were positive for *Chlamydia*, their titers were 2 to 3-fold lower compared to controls (Fig 4B). Due to intimate anatomical connection between the mesenteric lymph nodes (MLNs) and the GI tract, we considered that infection of the MLNs, caused by circulating infected cells might be a source of cecal *Chlamydia*. Analysis of MLN *Chlamydia* titers revealed that MLNs become infected, likely by circulating cells however, the low frequency of infection and low MLN titers do not support this notion. In IV-infected mice *Chlamydia* MLN titers peaked at 6 dpi and diminished by 9 dpi (S2A Fig). Similarly, in PV-infected mice MLN titers peaked at 7 dpi and show clearance dynamics similar to the spleen (S2B and S2C Fig and Fig 1). There were no significant differences in MLN *Chlamydia* titers among time points in IV-infected mice (p<0.08), or mice infected PV with 10^6^ (p<0.2) or 10^7^ (p<0.1) IFU of *Chlamydia* (S2A–S2C Fig). Moreover, *Chlamydia* is present in MLNs of CCR7^-/-^ mice only at 14 dpi and the frequency of *Chlamydia* MLN infection and MLN titers are lower compared to the frequency of infection and titers in the spleen (S2D Fig and Fig 2B). Similarly, MLNs of FTY720-treated mice were not infected (S2E Fig) and only 0/8 and 1/8 splenectomized mice had MLN titers at 7 and 14 dpi, respectively (S2F Fig). Interestingly, splenectomized mice exhibit *Chlamydia* titers in the liver (S1E Fig), indicating that in the absence of the spleen, *Chlamydia*-harboring cells migrate to the liver, which then contributes to the infection of the GI tract, although less effectively compared to the spleen. While 5/16 (about 30%) of splenectomized mice were positive for *Chlamydia* in liver and ceca (at 7 and 14 dpi) (Fig 4B, S2E Fig), the incidence of liver infection in controls with intact spleens was only about 1% (S1E Fig). There were no differences in *Chlamydia* titers in vaginal swabs of control and splenectomized mice at 0, 6, 9, or 12 dpi (Fig 4C), indicating that the lack of the spleen does not affect FRT *Chlamydia* clearance in this timeframe. We also examined the gallbladder and blood fractions (plasma and cells separately) for presence of infection and of 46 and 64 samples respectively, we found no samples that were positive for live *Chlamydia* (S1E Fig).

### Systemic dissemination of *Chlamydia* is essential for induction of *Chlamydia*-specific antibody response and does not depend on *Chlamydia* ascension to the upper FRT

**Fig 5:**
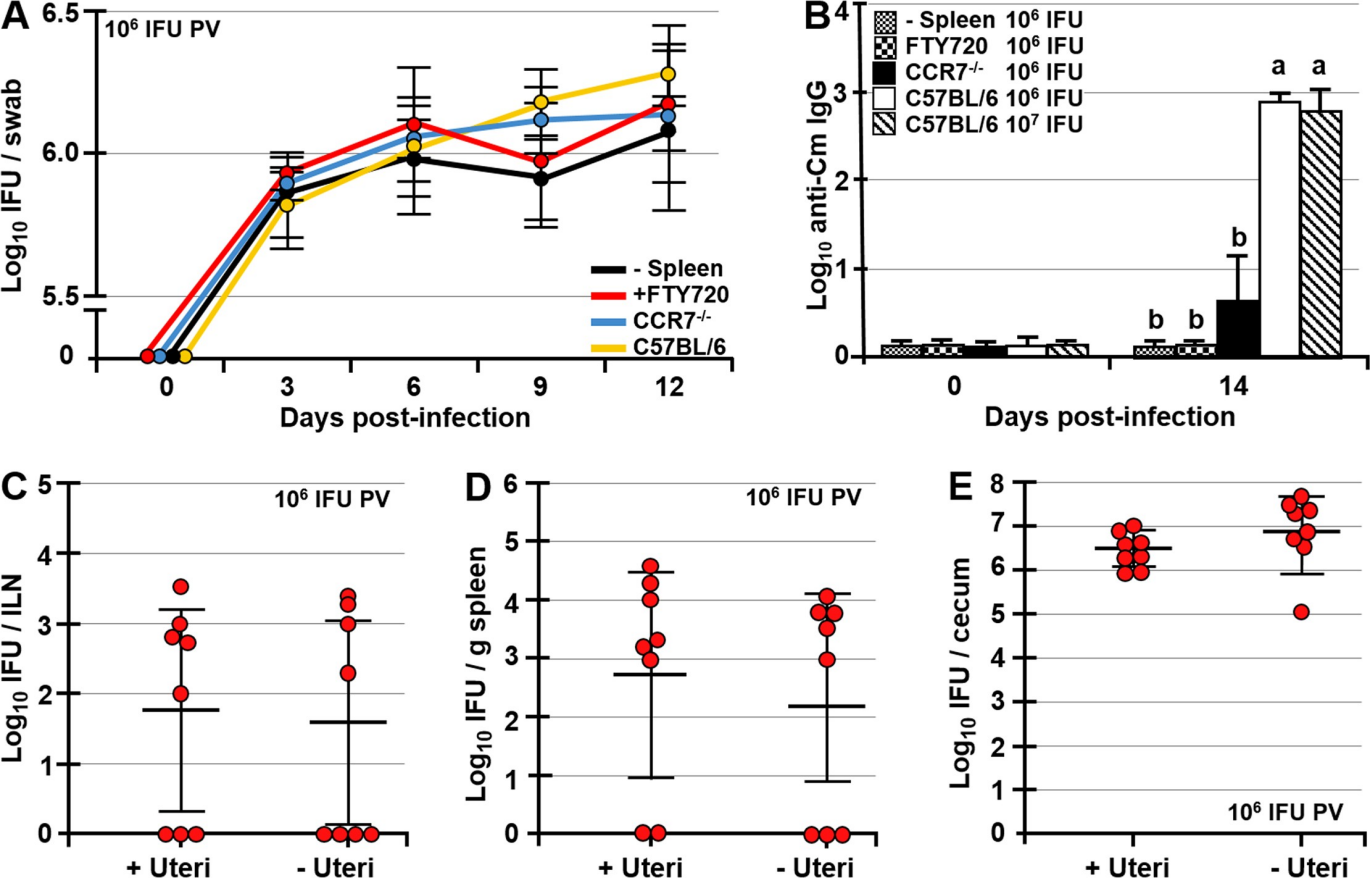
Systemic dissemination of *Chlamydia* is essential for induction of serum antibodies and does not depend on *Chlamydia* ascension in the upper FRT. (A) Vaginal swab titers of splenectomized, FTY720-treated, CCR7^-/-^, and C57BL/6 control mice at 0, 3, 6, 9 and 12 dpi PV with 10^6^ IFU of *Chlamydia*. (B) Serum IgG titers of splenectomized, FTY720-treated, CCR7^-/-^, and C57BL/6 mice at 14 dpi PV with 10^6^ (and 10^7^ controls) IFU of *Chlamydia*. IgG titers are expressed as log_10_ titer values, with the titer being the highest dilution having an absorbance value twice that of the background. (C-E) *Chlamydia* titers in ILNs, spleen and ceca of control and uterectomized mice at 7 dpi PV with 10^6^ IFU of *Chlamydia*. Data are representative of two experiments and are expressed as the mean ± SD. Group means were separated using Student’s t-test. Group means that do not share a superscript are significantly different from each other (p<0.05).

Although systemic dissemination of *Chlamydia* was inhibited in splenectomized, CCR7^-/-^, and FTY720-treated mice compared to controls (Figs 2–4), there were no differences in *Chlamydia* titers in vaginal swabs among these groups and controls at any time point (0, 3, 6, 9 or 12 dpi) (Fig 5A). Systemic dissemination of *Chlamydia* however, is critical for induction of adaptive immunity, as splenectomized, CCR7^-/-^, and FTY720-treated mice exhibited no (splenectomized, FTY720-treated), or significantly reduced (CCR7^-/-^) *Chlamydia*-specific serum IgG antibodies compared to C57BL/6 controls (Fig 5B). While all PV infected C57BL/6 mice had high serum IgG titers by 14 dpi regardless of the dose (10^6^ or 10^7^ IFU), all splenectomized and FTY720-treated mice were negative for *Chlamydia*-specific IgG, and only 3/11 CCR7^-/-^ mice had *Chlamydia*-specific IgG titers (albeit low), reflecting the observed delayed *Chlamydia* dissemination in CCR7^-/-^ mice (Fig 5B). In addition, ascension of *Chlamydia* in the upper FRT is not critical for its systemic dissemination, as there were no differences in *Chlamydia* titers in ILNs (p<0.4), spleen (p<0.3), or ceca (p<0.8) between controls and uterectomized mice (Fig 5C–5E) at 7 dpi.

### CD11c^+^ DCs transport *Chlamydia* from the FRT to the draining ILNs

**Fig 6:**
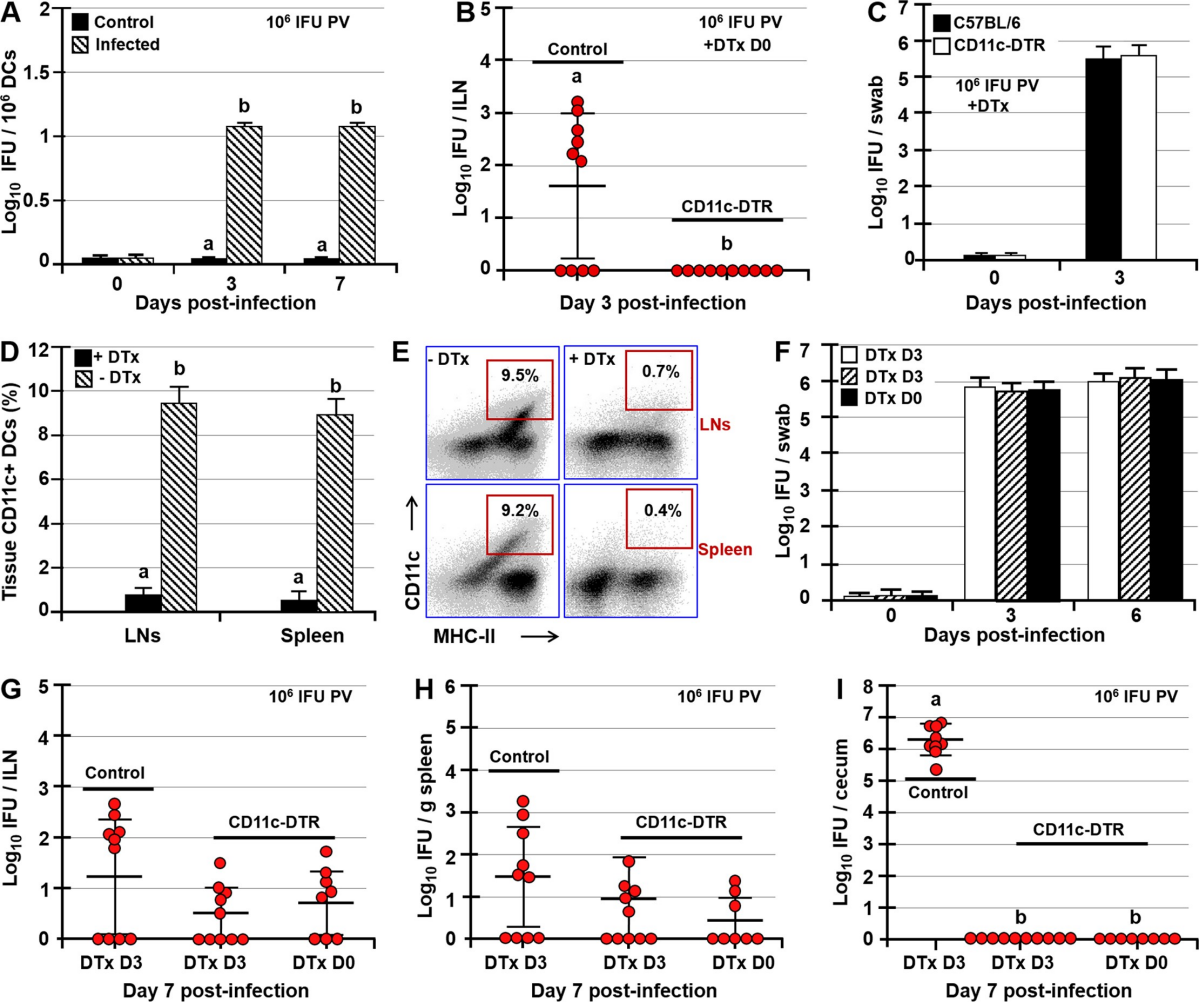
Dissemination of *Chlamydia* from the FRT to the draining ILNs depends on *Chlamydia* carriage by migrating CD11c^+^ DCs. (A) Recovery of *Chlamydia* from CD11c^+^ DCs isolated from ILNs of uninfected (control) and PV-infected mice at 0, 3, and 7 dpi PV with 10^6^ IFU of *Chlamydia*. (B) *Chlamydia* titers in ILNs and (C) vaginal swabs of C57BL/6 (control) and CD11c-DTR mice at 3 dpi following treatment with DTx at 0 dpi (n = 10 mice/group). (D, E) Flow cytometry analysis of CD11c^+^ DC proportions in LNs and spleens of CD11c-DTR mice at 24h post-treatment with PBS (control) or DTx. (F) Vaginal swab titers of control (C57BL/6, white bars) or CD11c-DTR mice PV infected with 10^6^ IFU of *Chlamydia* and treated with DTx at 0 or 3 dpi (black or striped bars, respectively). (G-I) *Chlamydia* titers at 7 dpi in ILNs (G), spleen (H), and ceca (I) of C57BL/6 (control) and CD11c-DTR mice treated with DTx at 0 or 3 dpi PV with 10^6^ IFU of *Chlamydia*. Data are representative of two experiments and are expressed as the mean ± SD. Group means were separated using Student’s t-test or Tukey’s multiple comparison procedure. Group means that do not share a superscript are significantly different from each other (p<0.05).

At 7 dpi there was a significant increase in the ILN size (S3A and S3B Fig), likely caused by induced immune responses, increased cell recruitment and proliferation following ILN infection. Moreover, live *Chlamydia* was present in CD11c^+^ DCs isolated from ILNs of PV-infected mice at 3 and 7 dpi (Fig 6A). We then set out to examine whether CD11c^+^ DCs of the FRT mediate *Chlamydia* transport from the FRT to the draining ILNs. To address this question, control (C57BL/6 background that lack high affinity diphtheria toxin (DTx) receptor) and CD11c-DTR mice were injected intra-peritoneally (IP) with DTx at 0 or 3 dpi for selective depletion of CD11c^+^ DCs. At 3 or 7 dpi ILNs, spleen, and cecal scrapings were collected and their *Chlamydia* loads determined. Depletion of CD11c^+^ DCs prior to infection completely abrogated infection of the ILNs at 3 dpi (Fig 6B) with ILNs of all 10 mice (across two separate studies) being negative for *Chlamydia* (Fig 6B). As expected, no *Chlamydia* was recovered from spleens and ceca of control or CD11c-DTR mice at 3 dpi (S3D and S3E Fig). These results indicate that CD11c^+^ DCs transport *Chlamydia* from the FRT to the ILNs (Fig 6B). In addition to being negative for *Chlamydia*, ILNs of CD11c-DTR mice were of normal size, much like the ILNs of control mice (S3A Fig). The lack of CD11c^+^ DCs did not affect *Chlamydia* loads at 3 dpi in vaginal swabs, as there were no differences in titers between control and CD11c-DTR mice (Fig 6C). Depletion of CD11c^+^ DCs was confirmed by FACS analysis of CD11c^+^ DC proportions in the lymph nodes and spleens of control and CD11c-DTR mice at 24h post-DTx treatment (Fig 6D and 6E). We then examined whether CD11c^+^ DC depletion prior to and after infection of ILNs (at 0 or 3 dpi) would affect *Chlamydia* burden in the spleen and the GI tract at 7 dpi. DTx treatment had no effect on FRT *Chlamydia* titers, as no differences in *Chlamydia* loads of vaginal swabs were observed among DTx-treated controls or CD11c-DTR mice at 3 (p<0.8) or 6 dpi (p<0.8) (Fig 6F). At 7 dpi, we saw no significant differences in *Chlamydia* titers in ILNs (p<0.18) and spleens (p<0.4) among C57BL/6 controls and CD11c-DTR mice following DTx treatment at 0 or 3 dpi, although CD11c-DTR mice had numerically lower *Chlamydia* loads in these tissues (Fig 6G and 6H). However, depletion of CD11c^+^ DCs at 0 or 3 dpi abrogated *Chlamydia* dissemination to the GI tract, as at 7 dpi ceca of all CD11c-DTR mice (treated with DTx at 0 or 3 dpi) were negative for *Chlamydia* and DTx-treated control mice exhibited significantly higher cecal *Chlamydia* titers (p<0.001) (Fig 6I). Presence of *Chlamydia* in spleens, but not ceca of DTx-treated CD11c-DTR mice at 7 dpi again indicates that *Chlamydia* infects the spleen before spreading to the GI tract. In addition, there were no differences in cecal *Chlamydia* titers between DTx-treated and untreated controls (S3C Fig). Taken together, our findings may be summed up as follows: 1) After FRT infection, *Chlamydia* reaches the GI tract in stages, by first infecting ILNs, then the spleen, and the GI tract; 2) Low dose PV infection delays, but does not abrogate *Chlamydia* dissemination to the spleen and the GI tract; 3) Ascension of *Chlamydia* to the upper FRT is not essential for its systemic dissemination; 4) Inhibition of cell migration in CCR7^-/-^ and FTY720-treated mice delays or abrogates *Chlamydia* systemic dissemination; and 5) CD11c^+^ DCs mediate the first step of *Chlamydia* systemic dissemination, from the FRT to the draining ILNs.

## Discussion

**Fig 7:**
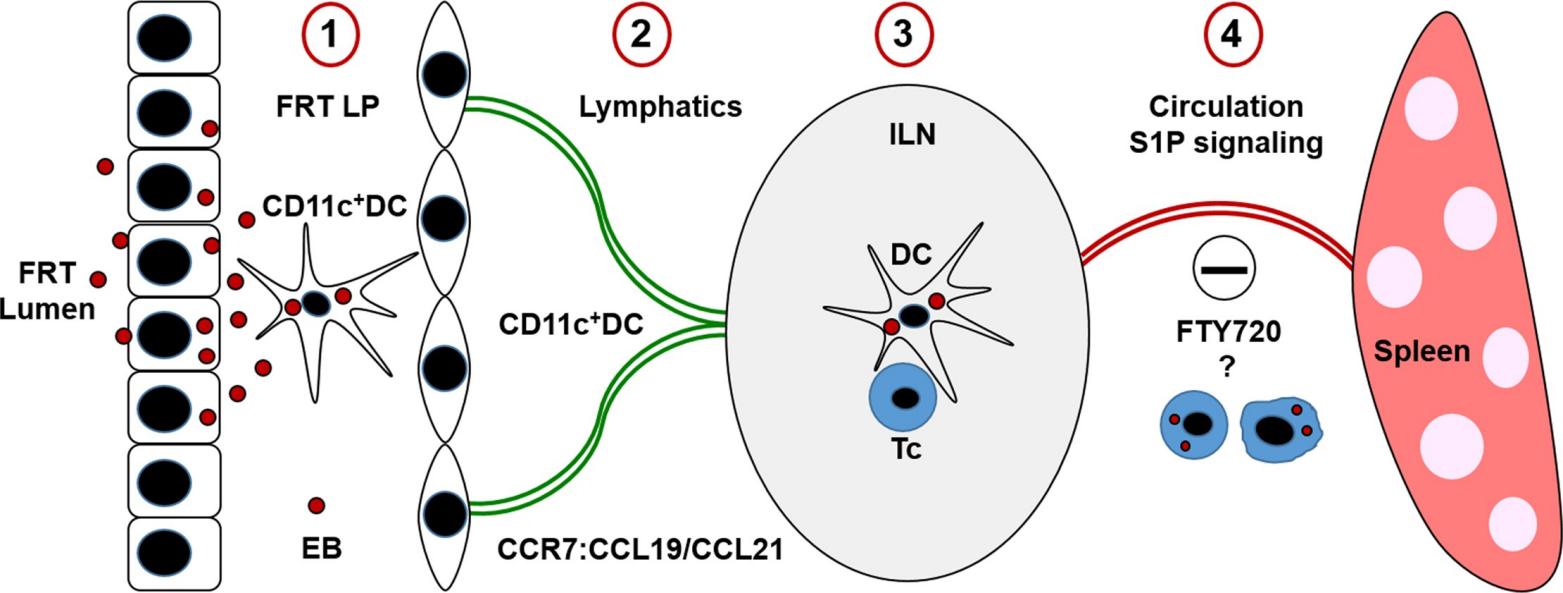
A working model of *Chlamydia* dissemination from the FRT to the GI tract. *Chlamydia* first infect epithelial cells of the FRT. CD11c^+^ DCs capture *Chlamydia* EBs in the lamina propria (LP) of the FRT, which triggers DC migration to the FRT-draining ILNs. In the ILNs, immune responses to *Chlamydia* are initiated, leading to lymphocyte activation, recruitment, and proliferation. The second step of *Chlamydia* transport from ILN to the spleen (by yet to be identified host cells) depends on S1P-mediated cell migration, which is inhibited by FTY720 treatment.

*C*. *trachomatis* is a human-specific pathogen that can cause long-term disease sequela such as PID, ectopic pregnancy, endometriosis, tubal fibrosis, and infertility [31]. The long-term FRT pathology (PID) occurs in about 10% of women, likely because infections remain undetected and untreated [4]. *C*. *trachomatis* is also routinely found in the GI tract of women. However, it remains unknown whether systemic dissemination of *C*. *trachomatis* plays a role in GI tract infection. In mice, under experimental settings in which coprophagy and grooming are precluded, *Chlamydia* disseminates from the FRT to the GI tract via an internal, non-oral, non-rectal route [22], although no mechanism of transit has been proposed. Blood-borne *C*. *muridarum* can establish a long-lasting infection in the GI tract, leading to the suggestion that *C*. *muridarum* disseminates via the circulation [23]. Since IV infection is not an appropriate model for PV *C*. *trachomatis* infection in women, we used the PV *C*. *muridarum* mouse model of infection. Using this model we show that *Chlamydia* dissemination from the FRT to the GI tract depends on active cell migration and consists of three distinct steps: 1) DC-mediated *Chlamydia* transport from the FRT to the draining ILNs; 2) S1P-dependent *Chlamydia* transport from ILNs to the spleen; and 3) *Chlamydia* transport from the spleen to the GI tract via yet to be determined mechanisms. Based on presented data we propose the following model of systemic *Chlamydia* dissemination (Fig 7). After initially infecting epithelial cells of the FRT, *Chlamydia* EBs are internalized by lamina propria CD11c^+^ DCs (1). This triggers DC maturation and increased expression of CCR7, which allows DCs to sense CCL21, enter local lymphatics (2) and migrate to the draining ILNs (3). In the ILNs, *Chlamydia* undergoes EB-RB-EB differentiation and proliferation. In the ILNs, other and yet to be identified cells become infected, enter the circulation by sensing the higher S1P gradients, and migrate to the spleen (4) where *Chlamydia* infects other cells and proliferates before being ferried to the GI tract.

DCs are potent antigen presenting cells and T cell activators that also play important roles in antigen capture and transport, cytokine secretion, and thus induction of immunity and tolerance [32–34]. Most evidence showing a significant role of mucosal DCs in pathogen uptake and transport has been gathered from HIV studies. In explanted human FRT and intestinal tissues, DCs capture and transport HIV through the mucosa [35, 36], leading to the hypothesis that DCs capture the virus and carry it to the lymph nodes resulting in the establishment of a systemic infection. Although HIV and *Chlamydia* spp. are very different pathogens, similar mechanisms appear to mediate their systemic spread following FRT infection. Depletion of CD11c^+^ DCs prior to infection abrogates *Chlamydia* spread to the ILNs, indicating that DCs carry *Chlamydia* to the ILNs, much like the proposed HIV transport by DCs. Although signaling cues that direct DC egress from the FRT during *Chlamydia* infection have not been previously examined, CCR7 signaling is a critical mediator of DC migration from the skin [37], intestinal mucosa [38] and the lungs [39]. The lack of FTY720 treatment effect in the FRT-ILN *Chlamydia* spread is in line with findings that immature DCs do not migrate to S1P [29]. Moreover, migration of DCs to draining lymph nodes in mice that lack S1P in lymphatic fluid is not different from DC migration in wild-type mice [40] which indicates that S1P signaling is dispensable for guiding FRT DC migration and thus *Chlamydia* transport to ILNs.

The decreased *Chlamydia* transport to ILNs in CCR7^-/-^ mice indicates that CCR7:CCL19/CCL21 signaling axis is critical for FRT to ILN DC migration. CCR7 chemokine agonist CCL21 is expressed in lymphatic endothelial cells and lymph nodes. It is thus essential for DC entry into local lymphatics and for DC migration to and within LNs [28, 41, 42]. Indeed, in CCR7^-/-^ mice tissue DC migration is reduced by about 90% under inflammatory conditions [27, 43], which may explain the delayed FRT to ILN *Chlamydia* spread. The delayed *Chlamydia* spread in CCR7^-/-^ mice may also indicate that in the absence of CCR7 other signaling receptor/chemokine pairs (such as CCR8:CCL1/CCL8, CXCR4/CXCL12 and CX3CR1/CX3CL1) direct DC migration [44–46]. The essentiality of CCR7 for migration of both DCs and Langerhans cells was confirmed in irradiated wild-type mice that were reconstituted with wild type and CCR7^-/-^ bone marrow. In this model, the expression of CCR7 in wild-type DCs conferred a strong competitive advantage for accumulation of migratory DCs in draining lymph nodes [47]. However, since CCR7^-/-^ mice exhibit anomalies in lymph node architecture and cell distribution [27], studies using bone marrow chimeras are warranted to confirm the definitive role of CCR7 signaling in FRT DC migration and *Chlamydia* transport.

Inhibition of the second step of *Chlamydia* transport, namely from the ILNs to the spleen by FTY720, indicates that S1P signaling is essential for egress of *Chlamydia*-infected cells from the ILNs. Within ILNs *Chlamydia* was recovered from isolated CD11c^+^ DCs at 3 and 7 dpi. Infected DCs may be of FRT origin, or may be local ILN DCs that become infected by incoming FRT DCs. DCs also appear to be a significant *Chlamydia* reservoir in the ILNs, as their depletion at 3 dpi lowers ILN and spleen *Chlamydia* loads and abrogates GI tract infection at 7 dpi. While there is abundant evidence that tissue DCs transport antigen to the local draining lymph nodes, there is no evidence that once in the lymph nodes, tissue DCs re-enter the circulation to reach distant sites, such as the spleen. Rather, once in the lymph nodes, tissue-migratory DCs lose their motility [48] and most die [49]. Other cell types that migrate towards S1P in vivo such as T cells, NK cells [50, 51], and monocytes [52] may also contribute to *Chlamydia* dissemination. However, DCs have been shown to pass HIV infection to T cells either directly via transfer of surface virions (trans-infection) [53] or via the exosomes [54]. It is thus possible that during antigen presentation in the ILNs DCs pass *Chlamydia* EBs to T lymphocytes, which then become effector T cells, enter the circulation in responses to higher S1P concentration and ferry *Chlamydia* initially to the spleen and then to the GI tract. Alternatively, EBs might not need to productively infect T cells, but merely bind to T cell surface receptors in order to be carried to the spleen and the GI tract. This possibility is in line with our finding that FTY720 inhibits ILN to spleen/GI tract, but not FRT to ILN *Chlamydia* spread. Our finding that removal of the spleen significantly diminishes *Chlamydia* infection of the GI tract provides additional support to this notion. Furthermore, our finding that DC depletion at 0 and 3 dpi does not preclude spleen infection at 7 dpi, but does preclude GI tract infection, indicates that infection of the spleen is an important step in *Chlamydia* dissemination to the GI tract.

We did consider the possibility that mesenteric lymph nodes (MLN) may be the source of GI tract *Chlamydia* and analyzed *Chlamydia* titers in MLNs of IV and PV-infected mice. Although MLNs can become infected (likely by circulating *Chlamydia*-infected cells), they do not appear to be the source of GI tract *Chlamydia*. Our finding that splenectomized and FTY-treated mice have lower *Chlamydia* titers in the ILNs at 14 dpi might indicate that recirculating infected effector T cells that develop during protective immune responses home to the sites of infection (ILNs), and thus might contribute to the maintenance of *Chlamydia* loads in the ILNs.

While inhibition of systemic *Chlamydia* dissemination in splenectomized, CCR7^-/-^, or FTY720-treated mice did not affect vaginal shedding (for up to 14 dpi), it significantly hindered the induction of *Chlamydia*-specific serum IgG. Women with tubal infertility present with high titers of *Chlamydia*-specific IgG antibodies [14, 55, 56] therefore this finding sheds an important light on the possible connection between systemic *C*. *trachomatis* dissemination and long-term FRT pathology in humans. In both mice and humans, CD4^+^ T cells are essential for the resolution of chlamydial infections. However, the role of *Chlamydia*-specific antibodies [31, 57] and CD8^+^ T cells remains unclear [58]. Depletion of CD8^+^ T cells was shown to significantly reduce hydrosalpinx and *Chlamydia* spread to the GI tract in mice, leading to the suggestion that the GI tract infection may promote hydrosalpinx by inducing “pathogenic” CD8^+^ T cells [59].

Based on current knowledge gathered from clinical and animal studies a “two-hit model” of chlamydial pathogenesis was proposed [60], according to which the 1^st^ hit is caused by ascension of *Chlamydia* to the upper FRT that leads to tissue damage and formation of MHC:*Chlamydia* peptide complexes. The 2^nd^ hit that results in long-term pathology of fibrosis and hydrosalpinx is then delivered by recruited “pathogenic” CD8^+^ T cells, induced in the GI tract following *Chlamydia* dissemination. Based on our findings described here, we propose a revised hypothesis, according to which the 1^st^ hit is the *Chlamydia* spread to the spleen, where *Chlamydia*-specific “pathogenic” CD8^+^ T cells are induced. The 2^nd^ hit occurs when *Chlamydia* reaches the upper FRT (by ascension or by recirculating infected cells), leading to recruitment of *Chlamydia*-specific CD8^+^ T cells, which contribute to the long-lasting FRT pathology.

This work will be important for understanding the signaling mechanisms that govern cell migration and antigen transport from the FRT during an infection. In addition, further elucidating in vivo *Chlamydia* cell tropism, as well as delineating the roles of lymphoid tissues (e.g. the spleen) in generation of “pathogenic” *Chlamydia*-specific CD8^+^ T cells, will be critical for identifying targets for therapeutic intervention and vaccine development.

## Materials and methods

### Cell and bacterial culture conditions

Human cervical carcinoma epithelial cells (HeLa 229, ATCC CCL-2) were grown at 37°C with 5% CO_2_ in Dulbecco’s modified Eagle medium (DMEM) that was supplemented with 10% fetal bovine serum (DMEM-10) and 50 μg/mL gentamicin. *Chlamydia muridarum* strain Nigg was propagated in HeLa cells and purified as described previously [61]. Briefly, confluent cell monolayers were infected by centrifugation for 1 h at 545 x *g*, or rocking for 2 h at 37°C and 5% CO_2_. Infected cells were then incubated in DMEM-10 supplemented with 1x nonessential amino acids, and 1 μg/mL cyclohexamide for 40 h, after which HeLa cells were ruptured by sonication and cell debris pelleted by centrifugation (500 x *g*). Collected supernatant harboring EBs was then centrifuged at 10,000 x *g*, following which pelleted EBs were collected and purified on Percoll gradient by centrifugation at 30,000 x *g*, as described previously [62]. All *Chlamydia* EB stocks were stored at -80°C in sucrose-phosphate buffered glutamic acid (SPG) until used.

### Animals

Female 6–8 week-old C57BL/6, CCR7^-/-^, and CD11c-DTR mice were purchased from the Jackson Laboratory (Bar Harbor, ME) and housed under specific pathogen free conditions.

### Ethics statement

Studies were conducted in strict accordance with recommendations of the Guide for the Care and Use of Laboratory Animals of the National Institutes of Health. The animal protocol (Protocol # 18–041) was approved by the Southern Illinois University Institutional Animal Care and Use Committee. Mice were anesthetized with 1–3% isoflurane delivered in a stream of oxygen by a controlled precision vaporizer. To ensure that animals were anesthetized, the respiration rate was monitored and a toe pinch was performed. At the end of the studies animals were euthanized using CO_2_. Cervical dislocation was additionally performed to ensure that the animal was deceased.

### Animal infections with *Chlamydia*

Mice were PV infected with either 10^3^, 10^4^,10^5^, 10^6^ or 10^7^ IFU of *Chlamydia* in a 10 μL volume of SPG. Five days prior to PV infections mice were sub-cutaneously (SC) injected with 2.5 mg medroxyprogesterone acetate (Henry Schein) in order to synchronize their estrus cycles. To exclude the possibility of *Chlamydia* transmission via coprophagy or grooming, mice were housed individually in wire-mesh bottom cages and fitted with custom-made, light-weight neck collars. In one study mice were infected IV with 10^5^ IFU of *Chlamydia* in 200 μl of sterile saline solution via a lateral tail vein. To consistently infect animals with intended doses of *Chlamydia*, a single stock of renographin-purified EBs with a known titer was used. At pre-determined times following PV or IV infections, mice were euthanized and tissues were collected for cell isolation, determination of *Chlamydia* titers, and *Chlamydia*-specific antibodies.

### Inhibition of cell egress from lymphoid tissues by FTY720

FTY720 (Sigma) was dissolved in drinking water (1.85 mg/L) supplied ad libitum to C57BL/6 mice 3 d prior to infection and for the reminder of the studies (for up to 14 dpi).

### Infection of HeLa cell monolayers with tissue homogenates

At 3, 7, and 14 d post PV or 3, 6 and 9 d post IV infection, mice were euthanized and blood was collected via cardiac puncture. Plasma and blood cells were separated by centrifugation. From each mouse at any given time point 4x10^6^ cells were resuspended in 400 μL of SPG, sonicated twice (for 5 sec) and cell debris pelleted by centrifugation. Collected supernatant was used to infect fresh HeLa monolayers (2 replicates, 200 μL each). For titering samples of plasma, HeLa monolayers were overlaid with 200 μl of plasma that was either undiluted, diluted 1:2, or 1:4 in SPG. ILNs, spleen, liver, gallbladder, MLNs and ceca were aseptically excised and placed in sterile tubes containing SPG. Liver and spleen tissue samples were weighed in order to determine *Chlamydia* titers per g of tissue. The ceca were dissected longitudinally and their contents were gently removed by washing with PBS containing 0.1 mg/mL gentamicin and 2.5 μg/mL Fungizone. Cecal epithelium was then gently scraped with a scalpel blade in SPG and deposited in a sterile Eppendorf tube [63]. Collected cecal scrapings, ILNs, liver, and spleen tissues were vortexed for 1 minute, homogenized, and then sonicated briefly. Cell debris was pelleted by centrifugation 425 x *g* for 5 min at 4°C. Collected supernatant was diluted in DMEM (1:2 ratio) and used to infect HeLa monolayers in 48-well plates in duplicate. At 40 h post-infection, bacteria were harvested and serial dilutions (10^−1^ to 10^−7^) were used to infect duplicates of confluent cell monolayers in 96-well plates by centrifugation for 1 h at 545 x *g* for titering. *Chlamydia*-negative cultures were expanded for six passages to ensure that very low *Chlamydia* titers could be detected. If no live *Chlamydia* were detected after six passages, tissues were considered *Chlamydia*-negative. All titers shown here are from the first passage and all samples that were negative in the first passage remained negative for *Chlamydia* for up to six passages.

### Titering of live *Chlamydia* in infected tissue homogenates, serum, and blood cell fractions

At 24 h post-infection monolayers were fixed with ice-cold methanol for 10 min at room temperature. Methanol was then aspirated and cells were incubated with an anti-*Chlamydia* monoclonal antibody conjugated to fluorescein at 37°C for 30 minutes (Pathfinder, *Chlamydia* Culture Confirmation System, BioRad). Cells were then washed 3 times with ddH_2_O and mounted with 90% glycerol. Fluorescent inclusions were counted at 400X magnification using a Leica DMIL microscope. For each replicate of each serial dilution, 20 random views were counted. The total number of live organisms in a given sample was calculated as described by others [22] and titers are expressed as log_10_ IFU per tissue (ILNs, MLNs), per g of tissue (spleen, liver), per vaginal swab, or per cecum.

### Determination of *Chlamydia*-specific antibody titers in sera using ELISA assay

At the end of each study (14 dpi) mice were euthanized and blood was collected by cardiac puncture. Flat-bottomed 96-well plates were coated with renografin-purified *Chlamydia* EBs at a protein concentration of 10 μg/ml in coating buffer (0.02 M Na_2_CO3/0.07 MNaHCO_3_ in H_2_O, pH 9.6). ELISA assays were conducted as described previously [64–67]. Antibody titers are expressed as log_10_ value of the highest reciprocal dilution that yielded an OD value twice that of a negative control.

### Surgical removal of the spleen and uteri

Mice were anesthetized with 1–3% isoflurane delivered in a stream of oxygen by a controlled precision vaporizer. To ensure that animals were anesthetized, the respiration rate was monitored and a toe pinch was performed before and during surgical procedures. Ophthalmic ointment was applied to the eyes of anesthetized animals using a sterile swab in order to prevent corneal drying. Preemptive analgesia (Meloxicam, 2 mg/kg body weight) was administered SC per recommendations of a veterinarian. The fur surrounding the surgical site was shaved using an electric razor and the surgical site was wiped free from hair with 70% ethanol. The skin was then disinfected with 3 applications of Betadine and 70% ethanol. For splenectomy the animal was placed on a surgical stage laying on its right side and draped with sterile gauze. The peritoneum was exposed by a 1–2 cm incision in the skin parallel to the edge of the rib cage, midway between the last rib and the hip joint. A second incision was made in the peritoneal wall, exposing the spleen. The spleen was exteriorized using forceps and the connective tissues were cut and blood vessels cauterized using a Bovie cautery pen in order to separate the spleen from the body. For removal of uteri a 2–2.5 cm ventral midline incision was made into the skin and then through the linea alba of the abdomen, exposing the FRT. The uterus and connected adipose tissue was exteriorized and the uterine horns and ovaries identified. A Bovie cautery pen was used to dissect through the mesometrial membrane and blood vessels to separate the uterus from the posterior body wall. The Bovie pen and/or small dissecting scissors were used to separate the uterine horn from the ovary, leaving the ovary and oviduct intact. A ligature using 5–0 absorbable suture was made at the base of each uterine horn and both uteri were severed anterior to the suture knot and removed from the body. Following uterectomy or splenectomy, the abdominal cavity was closed using a simple interrupted pattern with 5–0 absorbable polyglycolic acid suture. The skin incision was closed using 6–0 nylon suture in a simple interrupted pattern. Following surgery, topical antibiotic was applied to the incision daily and animals were fitted with light weight neck collars until the incision was fully healed (few days). Animals were allowed to recover for 2–3 weeks before being used for further studies.

### DC depletion and analysis of tissue cell suspensions by flow cytometry

CD11c-DTR mice and controls lacking the high-affinity DTR (C57BL/6 background) were IP injected with 4 ng/g body weight dose of DTx (Sigma) 10 h prior to PV infection (0 dpi) or 3 dpi with 10^6^ IFU of *Chlamydia*. At 3 or 7 dpi respectively, ILNs, MLNs, spleens and cecal scrapings were collected for determining *Chlamydia* titers. To confirm that CD11c^+^ DCs were depleted following DTx treatment, single cell suspensions of spleens and lymph nodes of control and CD11c-DTR mice (with and without DTx depletion) were analyzed by flow cytometry as described previously [68]. Cell suspensions were analyzed using monoclonal antibodies specific for CD11c and MHC class II (BioLegend).

### Determination of *Chlamydia* titers in CD11c^+^ DCs isolated from ILNs

At day 0, 3 or 7 post-PV infection with 10^6^ IFU of *Chlamydia*, single cell suspensions of ILNs isolated from infected and control mice were prepared and CD11c^+^ DCs were isolated using MACS MicroBeads kit (Miltenyi). Briefly, isolated DCs were sonicated on ice for 5 sec (2x) and cell debris was pelleted by centrifugation. Collected supernatant was used to infect HeLa monolayers for titering using Pathfinder as described above.

### Statistical analysis

Data were analyzed using ANOVA procedures and SAS software. Population means were separated using Tukey’s multiple comparison procedures or Student’s t-test and were declared significantly different at p<0.05. Data are expressed as the mean ± SD of the mean. As an alternative to Student’s t-test and in cases when normal distribution assumption was not met data were analyzed using Wicoxon/Kruskal-Wallis tests.

## Supporting information

S1 Fig Incidence of and titers of *Chlamydia* in tissues and vaginal swabs. (A-C) *Chlamydia* titers in ILNs, spleen and ceca of mice PV infected with 10^3^, 10^4^ or 10^5^ IFU of *Chlamydia*. (D) Vaginal swab titers of mice PV-infected with 10^3^, 10^4^, 10^5^, 10^6^ or 10^7^ IFU of *Chlamydia* at 0, 3, 6, 9 and 12 dpi. (E) Incidence of *Chlamydia*-positive samples of liver, gallbladder, blood plasma or cell fractions. (F) Vaginal swab titers of control and uterectomized mice at 0, 3, and 6 dpi. Data are expressed as the mean ± SD. Group means were separated using Tukey’s multiple comparison test or Student’s t-test and declared significantly different at p<0.05 (n = 5 (A-D) or n = 8 (F) mice per time point). Group means that do not share superscript are significantly different (p<0.05).
(TIF)

S2 Fig *Chlamydia* titers in mesenteric lymph nodes (MLNs) following IV or PV infection. (A-C) *Chlamydia* titers in MLNs at 0, 3, 6, and 9 or 0, 3, 7 and 14 dpi IV with 10^5^ (A) or PV (B, C) with 10^6^ or 10^7^ IFU of *Chlamydia*. (D-F) *Chlamydia* titers in MLNs of CCR7^-/-^, FTY720-treated, and splenectomized mice at 7 and 14 dpi PV with 10^6^ IFU of *Chlamydia*. (G) MLN *Chlamydia* titers at 7 dpi in mice treated with DTx at 0 or 3 dpi PV with 10^6^ IFU of *Chlamydia*. Data are expressed as the mean ± SD. Group means were separated using Tukey’s multiple comparison test and declared significantly different at p<0.05 (n = 5 mice per time point (A-C), or n = 8–10 mice per time point from two separate studies (D-G). Group means that do not share superscript are significantly different (p<0.05).
(TIF)

S3 Fig *Chlamydia* titers in C56BL/6 mice and CD11c-DTR mice with or without DTx treatment. (A, B) ILNs of control (A) or *Chlamydia*-infected (B) mice at 7dpi PV with 10^6^ IFU of *Chlamydia*. (C) Cecal *Chlamydia* titers in control C57BL/6 mice with or without DTx treatment. (D, E) *Chlamydia* titers at 3 dpi PV with 10^6^ IFU of *Chlamydia* in spleen and ceca of control and CD11c-DTR mice treated with DTx at 0 dpi.
(TIF)
