# Supplementary figures and images for: Dissemination of Chlamydia from the reproductive tract to the gastro-intestinal tract occurs in stages and relies on Chlamydia transport by host cells
Source: PLoS Pathog. 2019 Dec 2;15(12):e1008207. doi: 10.1371/journal.ppat.1008207 (PMC6907867; doi:10.1371/journal.ppat.1008207)

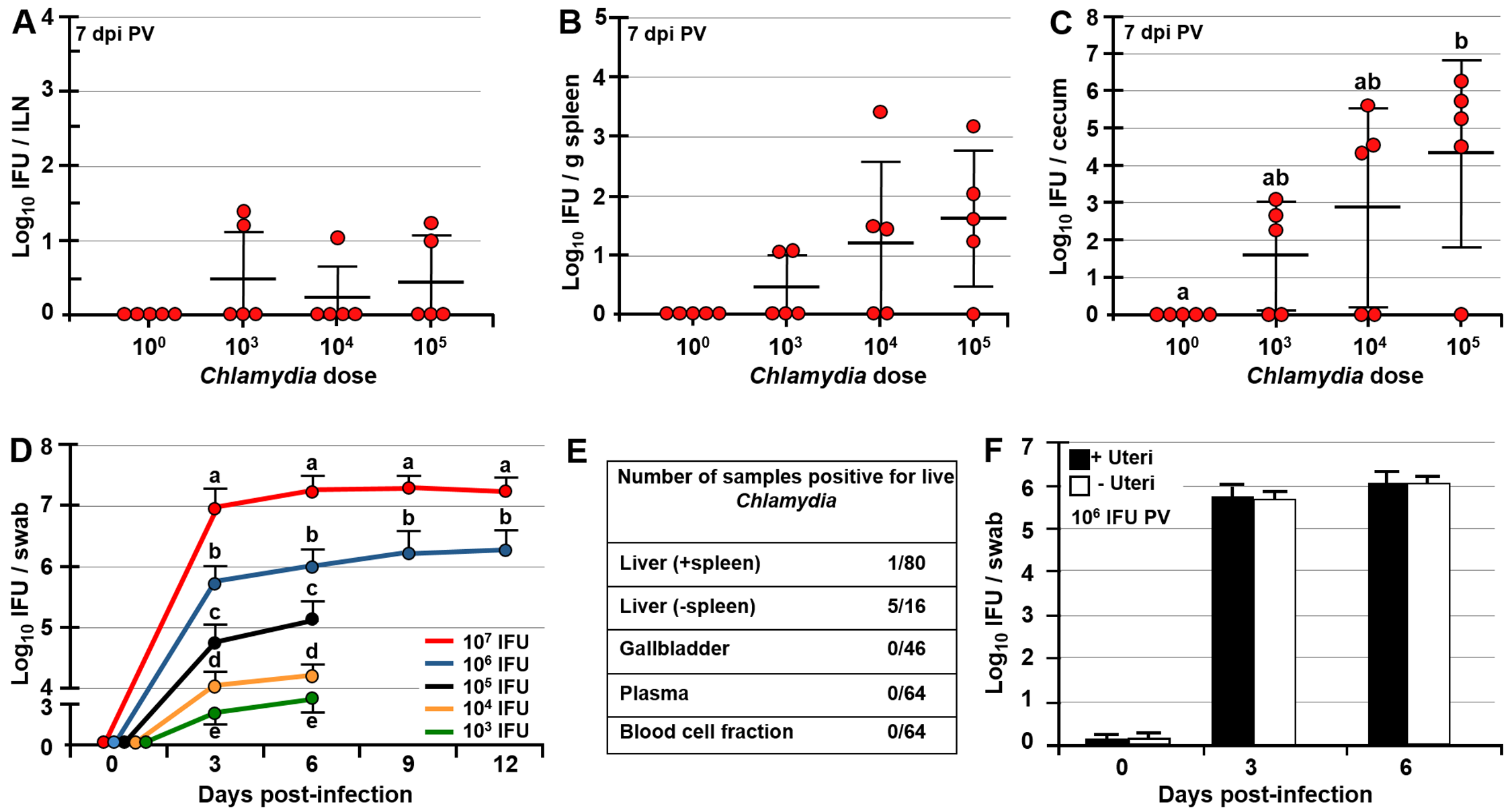

Supplement: S1 Fig — (A-C) Chlamydia titers in ILNs, spleen and ceca of mice PV infected with 103, 104 or 105 IFU of Chlamydia. (D) Vaginal swab titers of mice PV-infected with 103, 104, 105, 106 or 107 IFU of Chlamydia at 0, 3, 6, 9 and 12 dpi. (E) Incidence of Chlamydia-positive samples of liver, gallbladder, blood plasma or cell fractions. (F) Vaginal swab titers of control and uterectomized mice at 0, 3, and 6 dpi. Data are expressed as the mean ± SD. Group means were separated using Tukey’s multiple comparison test or Student’s t-test and declared significantly different at p<0.05 (n = 5 (A-D) or n = 8 (F) mice per time point). Group means that do not share superscript are significantly different (p<0.05). (TIF) [file ppat.1008207.s001.tif]

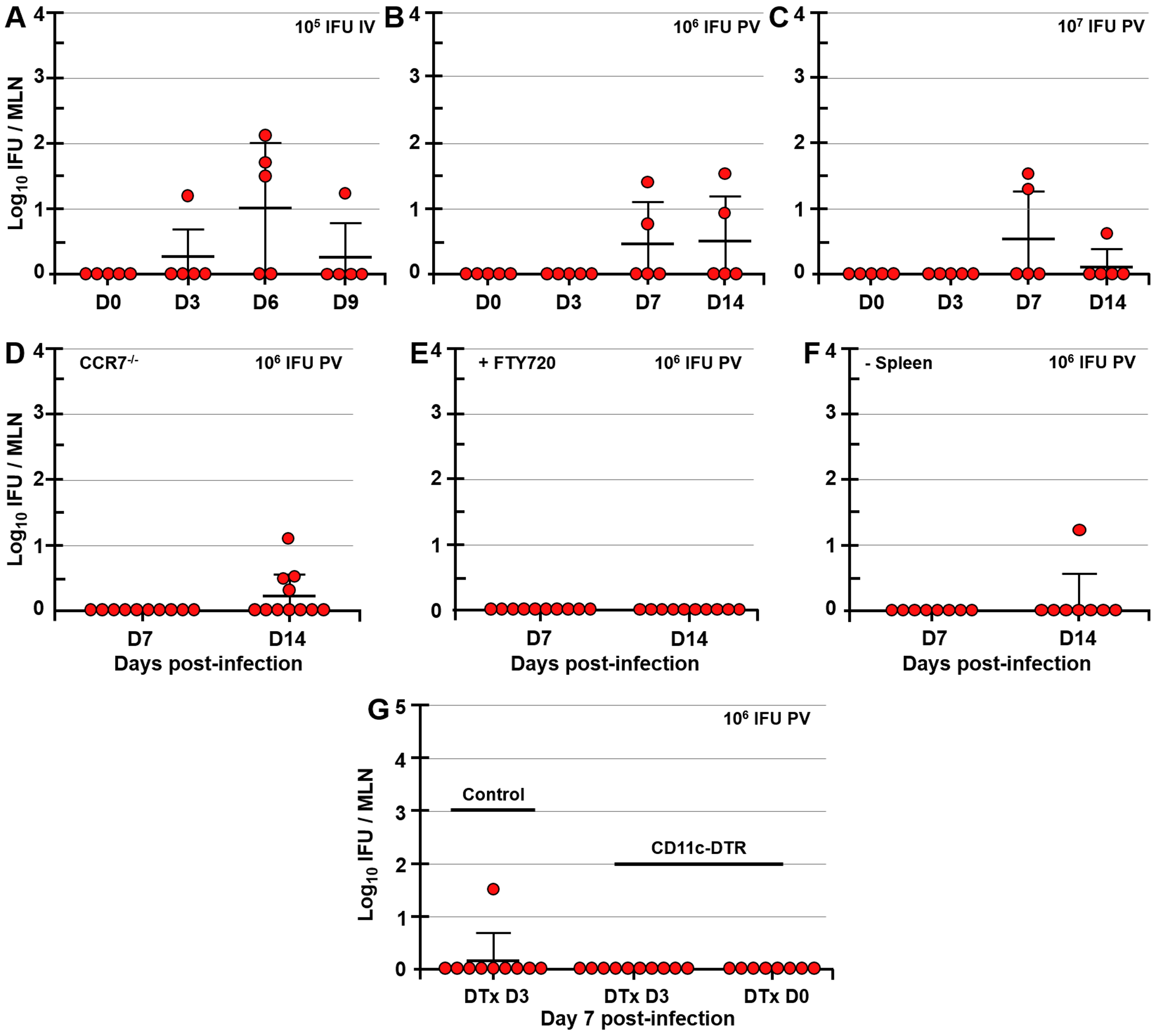

Supplement: S2 Fig — (A-C) Chlamydia titers in MLNs at 0, 3, 6, and 9 or 0, 3, 7 and 14 dpi IV with 105 (A) or PV (B, C) with 106 or 107 IFU of Chlamydia. (D-F) Chlamydia titers in MLNs of CCR7-/-, FTY720-treated, and splenectomized mice at 7 and 14 dpi PV with 106 IFU of Chlamydia. (G) MLN Chlamydia titers at 7 dpi in mice treated with DTx at 0 or 3 dpi PV with 106 IFU of Chlamydia. Data are expressed as the mean ± SD. Group means were separated using Tukey’s multiple comparison test and declared significantly different at p<0.05 (n = 5 mice per time point (A-C), or n = 8–10 mice per time point from two separate studies (D-G). Group means that do not share superscript are significantly different (p<0.05). (TIF) [file ppat.1008207.s002.tif]

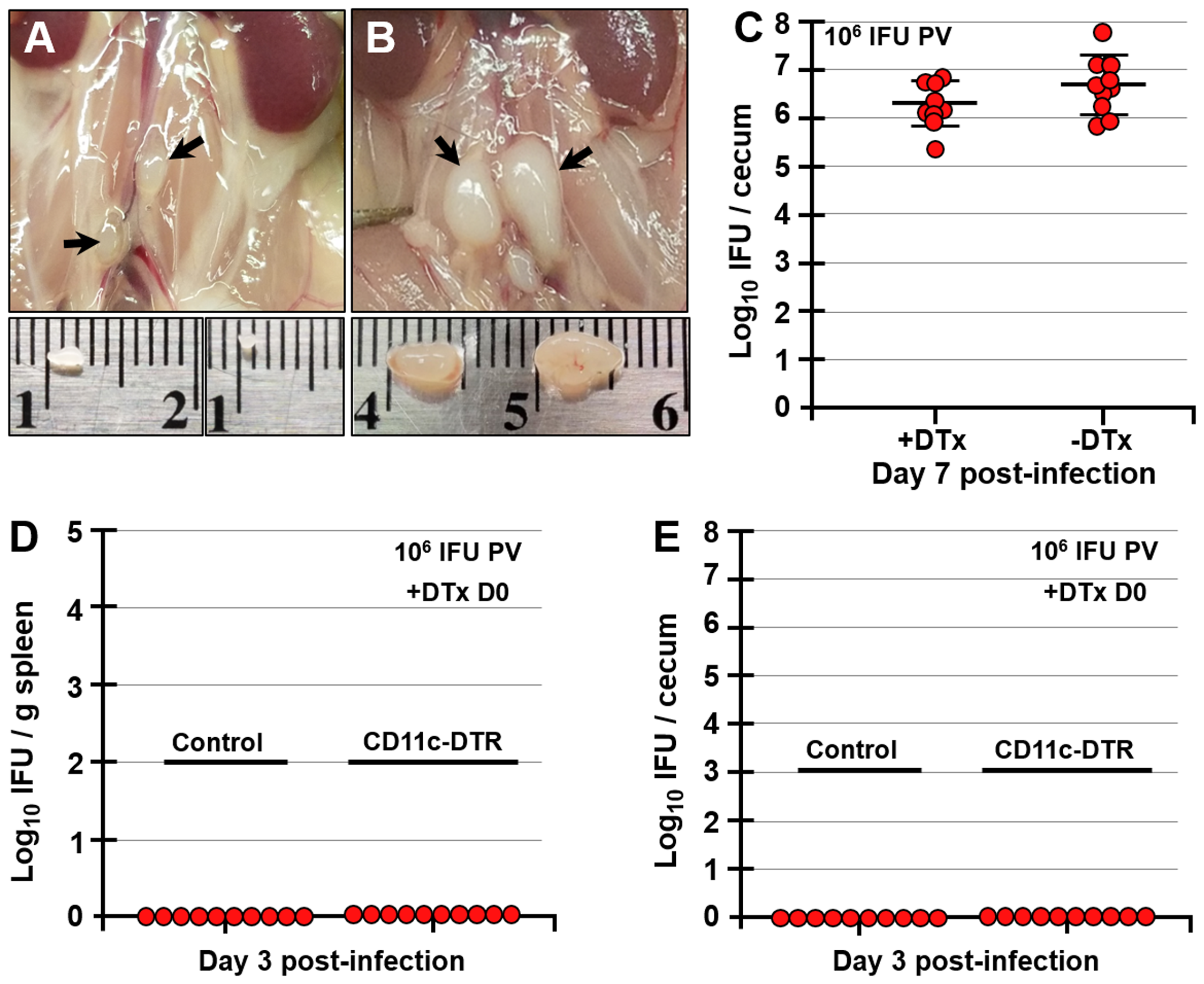

Supplement: S3 Fig — (A, B) ILNs of control (A) or Chlamydia-infected (B) mice at 7dpi PV with 106 IFU of Chlamydia. (C) Cecal Chlamydia titers in control C57BL/6 mice with or without DTx treatment. (D, E) Chlamydia titers at 3 dpi PV with 106 IFU of Chlamydia in spleen and ceca of control and CD11c-DTR mice treated with DTx at 0 dpi. (TIF) [file ppat.1008207.s003.tif]
